# Remodeling of the Nuclear Envelope and Lamina during Bovine Preimplantation Development and Its Functional Implications

**DOI:** 10.1371/journal.pone.0124619

**Published:** 2015-05-01

**Authors:** Jens Popken, Alexander Graf, Stefan Krebs, Helmut Blum, Volker J. Schmid, Axel Strauss, Tuna Guengoer, Valeri Zakhartchenko, Eckhard Wolf, Thomas Cremer

**Affiliations:** 1 Division of Anthropology and Human Genetics, Biocenter, LMU Munich, Planegg-Martinsried, Germany; 2 Chair for Molecular Animal Breeding and Biotechnology, Gene Center, LMU Munich, Munich, Germany; 3 Laboratory for Functional Genome Analysis (LAFUGA), Gene Center, LMU Munich, Munich, Germany; 4 Institute of Statistics, LMU Munich, Munich, Germany; 5 Division of Genetics, Biocenter, LMU Munich, Planegg-Martinsried, Germany

## Abstract

The present study demonstrates a major remodeling of the nuclear envelope and its underlying lamina during bovine preimplantation development. Up to the onset of major embryonic genome activation (MGA) at the 8-cell stage nuclei showed a non-uniform distribution of nuclear pore complexes (NPCs). NPCs were exclusively present at sites where DNA contacted the nuclear lamina. Extended regions of the lamina, which were not contacted by DNA, lacked NPCs. In post-MGA nuclei the whole lamina was contacted rather uniformly by DNA. Accordingly, NPCs became uniformly distributed throughout the entire nuclear envelope. These findings shed new light on the conditions which control the integration of NPCs into the nuclear envelope. The switch from maternal to embryonic production of mRNAs was accompanied by multiple invaginations covered with NPCs, which may serve the increased demands of mRNA export and protein import. Other invaginations, as well as interior nuclear segments and vesicles without contact to the nuclear envelope, were exclusively positive for lamin B. Since the abundance of these invaginations and vesicles increased in concert with a massive nuclear volume reduction, we suggest that they reflect a mechanism for fitting the nuclear envelope and its lamina to a shrinking nuclear size during bovine preimplantation development. In addition, a deposit of extranuclear clusters of NUP153 (a marker for NPCs) without associated lamin B was frequently observed from the zygote stage up to MGA. Corresponding RNA-Seq data revealed deposits of spliced, maternally provided *NUP153* mRNA and little unspliced, newly synthesized RNA prior to MGA, which increased strongly at the initiation of embryonic expression of *NUP153 *at MGA.

## Introduction

Recently, we observed a massive reorganization of nuclear architecture in bovine preimplantation embryos generated either by *in vitro* fertilization (IVF) or by somatic cell nuclear transfer (SCNT) [1,2]. These changes were most prominent during the transit through minor and major genome activation (mGA and MGA) which occur at the 2-cell stage [3] and at the 8-cell stage [4], respectively. Embryonic nuclei in both IVF and cloned embryos adopted a peripheral localization of chromosome territories (CTs) separated from each other by wide interchromatin (IC) channels, whereas their interior was occupied by a major lacuna deprived of chromatin, but enriched with splicing factors and newly synthesized RNA. Nuclei with this phenotype were termed ENPs (for Embryonic Nuclei with Peripheral chromatin arrangement) and were predominant at MGA. After MGA, CTs were redistributed throughout the nucleus and showed features of nuclear architecture typical for somatic cell types. This conventional nuclear phenotype is characterized by a layer of densely DAPI-stained chromatin beneath the nuclear envelope and around nucleoli, a non-random radial chromatin arrangement with gene-poor chromatin enriched in the nuclear periphery and gene-rich chromatin towards the nuclear center [5–8]. Nuclei with this phenotype were termed ENCs (for Embryonic Nuclei with a Conventional architecture).

The present study was initiated to explore changes of the nuclear envelope and its underlying lamina during these massive changes of the nuclear architecture in bovine preimplantation embryos. The nuclear lamina is located directly beneath the nuclear envelope and is made up of lamins B1 and B2 in developing cells and of lamins A and C in differentiated cells [9]. It serves as an anchoring matrix for chromatin with major implications for chromosome organization and gene expression [10]. Nuclear pore complexes (NPCs) are made up of eight clusters of about 30 nucleoporins (NUPs) [11] building a short tunnel with an inner diameter of ~50 nm [12] and an outer diameter of about 105 nm [13] to 145 nm [14] between a cytoplasmic and a nuclear ring spanning from the outer to the inner membrane of the bi-layer lipid nuclear envelope [15]. Eight fibers connect the nuclear ring with a more interior terminal ring forming the nuclear basket [16]. The 153 kDa nucleoporin NUP153 [17] can be localized at the nuclear ring and at the nuclear basket [18]. Chromatin can be associated with the nuclear basket and with internuclear filaments connected to the terminal ring [16].

Multiple, tubular, double-membraned invaginations of the nuclear envelope carrying NPCs were previously described in nuclei of a wide variety of species, including mammals, and it has been suggested that such invaginations would allow for close topographical relationships between NPCs and chromatin, as well as nucleoli, located in the nuclear interior [19]. In a recent review such invaginations were referred to as type II [20]. In addition, single-membraned invaginations, likely derived from the inner nuclear membrane and lacking NPCs, were described as type I [20]. Patterns of type I and type II invaginations can coexist in the same nucleus with branched type I extensions expanding from type II invaginations forming a nucleoplasmic reticulum (NR), which helps to perform export-import functions of the nuclear envelope not only in the nuclear periphery, but also deep within the nucleus [20].

We performed two-color immunostaining of NUP153 (as a marker for NPCs) and lamin B (as a marker for the lamina) in bovine embryos and recorded light optical serial sections from embryonic nuclei with structured illumination microscopy (3D-SIM). This approach yields a lateral resolution of about 100 nm and an axial resolution of about 300 nm. With conventional confocal laser scanning microscopy (CLSM) the corresponding resolution limits are 200 nm and 600 nm, respectively [21,22]. Accordingly, 3D-SIM provides roughly an eight-fold improvement of the volumetric resolution. Using this novel experimental approach we studied bovine embryos during preimplantation development with the main focus on changes in the nuclear envelope, its underlying lamina and the distribution of NPCs.

## Materials and Methods

### Ethics statement

Bovine oocytes for *in vitro* fertilization were obtained from cows slaughtered for meat production at the slaughterhouse Münchner Schlachthof Betriebs GmbH, Munich, Germany in compliance with EU regulations. No animal experiments were conducted.

### *In vitro* fertilization and cultivation of early bovine embryos

*In vitro* fertilization of bovine oocytes was performed as described in [23]. Cumulus-oocyte complexes (COCs) were obtained by aspiration from ovaries of slaughtered cows. COCs were matured in modified Parker’s medium consisting of TCM 199 supplemented with 5% estrous cow serum (ECS) and 0.2 U/ml o-FSH (Ovagen; ICPbio) for 20–22 hours at 39°C in humidified air with 5% CO_2_. Matured COCs were washed with the fertilization medium Tyrode's albumin lactate pyruvate (FERT-TALP) supplemented with sodium pyruvate (2.2 mg/ml), heparin sodium salt (2 mg/ml) and bovine serum albumin (BSA; 6 mg/ml) and transferred to 400-μl droplets of medium. Frozen spermatozoa were thawed at 38°C. 100 μl thawed sperm suspension covered by 1 ml capacitation medium was subjected to the swim-up procedure for 60 min. The COCs and spermatozoa (2 x 10^6^ cells/ml) were co-incubated for 18 hours at 39°C in humidified air with 5% CO_2_. Presumptive zygotes were mechanically denuded by vortexing, washed 3x in SOF culture medium enriched with 5% ECS, BME 100x (20 μl/ml; Invitrogen) and MEM (Minimum Essential Medium) 100x (10 μl/ml, Invitrogen) and transferred to 400-μl droplets of medium covered with mineral oil. Embryos were grown at 39°C in a humidified atmosphere of 5% CO_2_, 5% O_2_, and 90% N_2_ until they reached the appropriate stage for fixation.

### Fixation of embryos

Unless noted otherwise fixation of embryos and all subsequent steps were performed at room temperature. Embryos were briefly washed in 38°C PBS, fixed in 2% paraformaldehyde (PFA) in phosphate buffered saline (PBS), washed twice in PBS and then stored at 4°C in PBS until further use.

### Immunostaining and embedding

Background caused by PFA was quenched using 20 mM glycine in PBS for 10 min. After washing twice with PBS embryos were permeabilized with 0.5% Triton-X 100 for 15–30 min. After washing twice with PBS unspecific background signals were reduced by incubation in 2% BSA for 2 hours. Embryos were sequentially incubated in 40 μl of primary and secondary antibody solutions, diluted as specified in Tables 1 and 2 in PBS with 2% BSA. Specimens were incubated with primary antibodies overnight at 4°C. After washing 5x in PBS with 2% BSA the appropriate secondary antibodies, diluted in PBS with 2% BSA, were applied for 1 hour, again followed by 5x washing in PBS with 2% BSA and 5x washing without BSA. Thereafter fixation of antibodies was performed with 4% PFA in PBS for 10 min, followed by washing twice in PBS. Before the removal of the zona pellucida chromatin was counterstained with DAPI (4′,6-diamidino-2-phenylindole; Catalog No. D1306, Life Technologies) diluted in PBS (2.5 μg/ml) for 10 min followed by washing twice in PBS. Embryos or individual blastomeres were attached to precision cover glasses (18 mm x 18 mm; 170 ± 5 μm; Carl Roth, Germany; LH22.1) in PBS and embedded in Vectashield (Vector Laboratories).

**Table 1:** Primary antibodies.

Host	Target	Official name	Dilution	Company	ID
Goat	Epitope at C-terminus of Lamin B1. Detecting Lamin B1 and, to a lesser extent, Lamin B2	Lamin B Antibody (M-20)	1:100	Santa Cruz	SC-6217
Mouse	NUP153	Anti-NUP153 [QE5] antibody	1:200	Abcam	ab24700

**Table 2:** Secondary antibodies.

Host	Target	Fluorophore	Excitation [nm]	Dilution	Company	ID
Donkey	Mouse IgG (H+L)	Alexa	488	1:400	Invitrogen	A-21202
Donkey	Mouse IgG (H+L)	Alexa	594	1:500	Invitrogen	A-21203
Donkey	Goat IgG (H+L)	Alexa	488	1:400	Invitrogen	A-11055
Donkey	Goat IgG (H+L) preadsorbed	Alexa	594	1:400	Abcam	ab150136

### Three-dimensional confocal laser scanning microscopy (3D-CLSM)

3D-CLSM was performed using a high aperture, oil immersion objective (63x/1.4 NA). If the working distance of this objective was insufficient an objective with a lower aperture and a longer working distance (20x/0.7 NA) was used. Light optical serial sections of nuclei were recorded with a Leica TCS SP5 using x,y/z voxel sizes of 30–120 nm/200 nm for imaging of selected nuclei. Fluorochromes were excited with blue diode, argon and helium–neon lasers using laser lines at 405 nm, 488 nm and 594 nm respectively.

### 3D structured illumination microscopy (3D-SIM) and quantitative image evaluation

3D-SIM of embryonic nuclei was performed on a DeltaVision OMX V3 system (Applied Precision Imaging/GE Healthcare) with a lateral (x,y) resolution of ~120 nm and an axial (z) resolution of ~300 nm [7]. The system was equipped with a 100x/1.40 NA PlanApo oil immersion objective (Olympus), Cascade II:512 EMCCD cameras (Photometrics) and 405, 488 and 593 nm diode lasers. Image stacks were acquired with a z-distance of 125 nm and with 15 raw SIM images per plane (5 phases, 3 angles). The SI raw data were computationally reconstructed with channel specific measured OTFs using the softWoRX 4.0 software package (Applied Precision). Images from the different color channels were registered with alignment parameters obtained from calibration measurements with 0.2 μm diameter TetraSpeck beads (Invitrogen). The voxel size of the reconstructed images is 39.5 nm in x,y and 125 nm in z with 32-bit depth. For all subsequent image processing and data analysis, images were converted to 16-bit composite tif-stacks. Image stacks were processed using ImageJ 1.45b (http://rsb.info.nih.gov/ij/). Images are shown after application of a threshold, which removed background, including patterns apparently resulting from SIM imaging/reconstruction [1]. DAPI intensity classes were established as described [7]. The statistical comparisons were performed with the software package for scientific computing R 2.15 (http://www.r-project.org/).

### RNA-Seq data analysis

Bovine *in vitro* fertilized embryos were produced as previously described [24] using commercially available semen from a Zebu bull. Visually inspected embryos were picked and snap-frozen in liquid nitrogen after washing in PBS. Denuded oocytes before and after maturation and embryos at the 4-cell, 8-cell, 16-cell and blastocyst stages were collected for sequencing. In brief, pools of 10 oocytes or embryos were thawed and lysed in 10 μl of lysis buffer (Prelude kit from NuGEN) and cDNA was generated and amplified with the Ovation RNAseq V2 kit (NuGEN) following the instructions of the manufacturer and as described in [4]. For each stage three replicates were sequenced on an Illumina GAIIx in single-read mode and a read length of 80 bases. Data are available at Gene Expression Omnibus (GEO; GSE52415). Reads were analyzed as described in [4]. Briefly, reads were filtered from the 3' and 5' end with a quality cut-off of 20 and a minimal read length of 30. After filtering, reads were mapped with TopHat2 against the bovine reference genome (UMD 3.1) and the number of reads mapped to intronic positions of the genes *LMNA*, *LMNB1*, *LMNB2*, *NUP35*, *NUP37*, *NUP43*, *NUP50*, *NUP54*, *NUP62*, *NUP85*, *NUP88*, *NUP93*, *NUP98*, *NUP107*, *NUP133*, *NUP153*, *NUP155*, *NUP160*, *NUP188*, *NUP205*, *NUP210*, *NUP210L* and *NUP214* were counted. Reads in intronic regions vs. not-covered positions in intronic regions (RINP) values were calculated as previously described [4]. For the gene expression analysis, the variance stabilized expression values were obtained from the Bioconductor package DESeq.

## Results

In our study we combined DAPI staining of DNA with indirect immunostaining of NUP153 (as a marker for the presence of NPCs) and lamin B (as a marker for the nuclear lamina). If not stated otherwise, 3D-SIM was employed to obtain super-resolution light optical serial sections of nuclei.

### Nuclear pore complexes (NPCs) show profound changes of distribution patterns during preimplantation development

**Fig 1:**
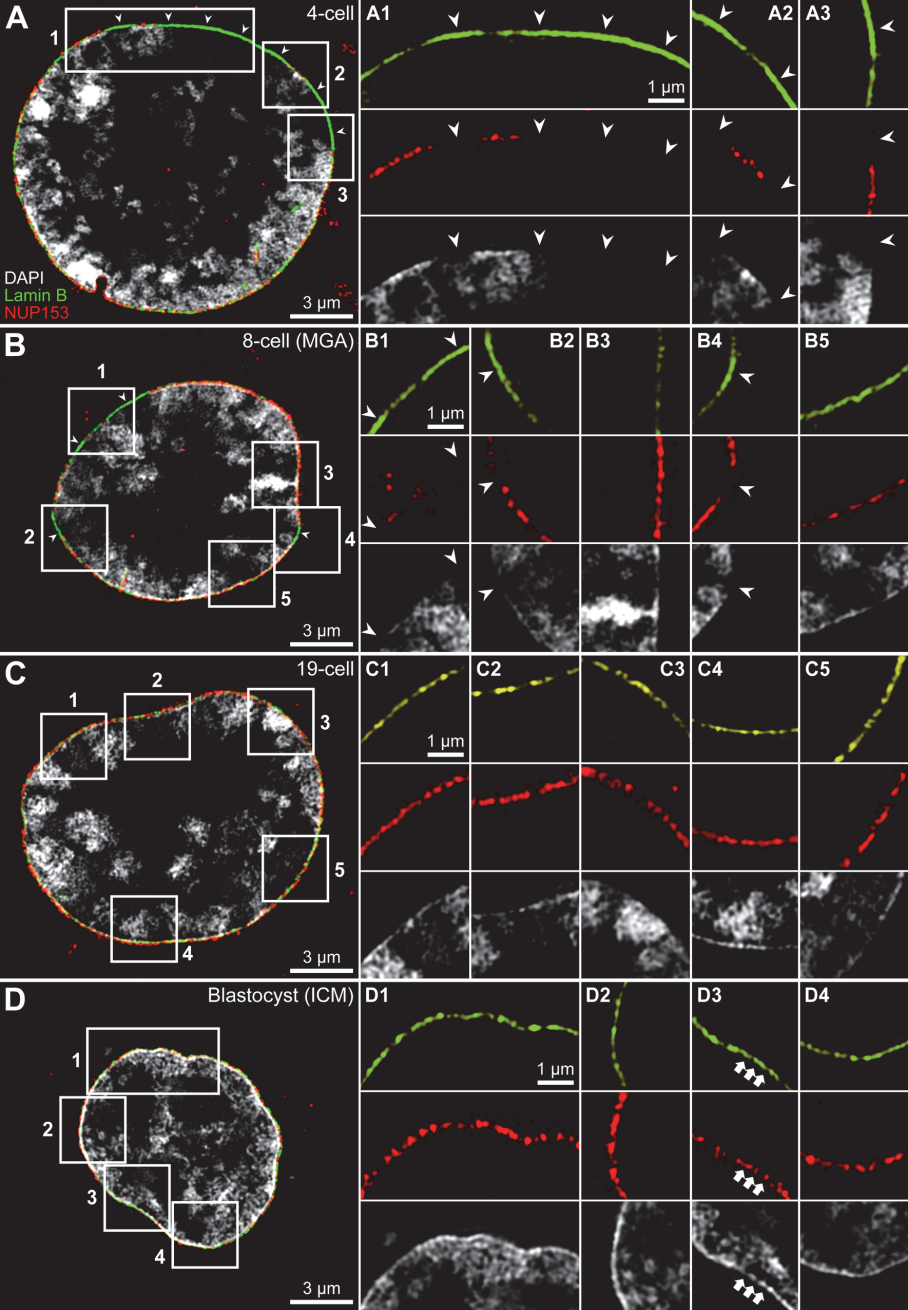
Presence of NPCs in the nuclear envelope correlates with chromatin contact sites. **A** and **B.** Midsections of nuclei from a 4-cell embryo (A) and an 8-cell embryo (B) demonstrate a lack of NUP153 signals (red) in regions of the nuclear envelope and associated lamin B (green) without contacts to DAPI-stained DNA (grey). **Panels A1-A3** and **B1-B5** show enlargements of boxed areas in A and B; arrowheads point to lamin B immunopositive, although NUP153 immunonegative regions without demonstrable DAPI-stained DNA. In contrast, the nuclear envelope carries NUP153 signals in regions, where the underlying lamina is contacted by DAPI-stained DNA. **C** and **D.** Midsections of nuclei from a 19-cell embryo (C) and from the inner cell mass of a blastocyst (D) reveal NUP153 signals distributed along the entire nuclear envelope. Enlargements of boxed areas shown in panels **C1-C5** and **D1-D4** demonstrate a strict correlation between contacts of DAPI-stained DNA along the entire lamina and the presence of NPCs. Note that DAPI-stained DNA indicates that the lamina is contacted by extended chromatin masses at some sites, whereas at other sites only a thin lining of chromatin can be noted beneath the lamina. This thin chromatin layer and the associated lamina are perforated by IC channels (D3, arrows). S1 Fig, panels D-F and H, present complete DAPI-, lamin B- and NUP153-stained sections of the nuclei shown in this Figure. Bars: 3 μm for A-D; 1 μm for A1-A3, B1-B5, C1-C5, D1-D4.

We demonstrate massive changes of nuclear phenotypes and nuclear pore distributions during different stages of preimplantation development (Fig 1; S1 and S2 Figs). Whereas the lamina showed a contiguously immunopositive staining for lamin B in nuclei at all stages, the distribution of NPCs was strikingly inhomogeneous in the nuclear envelope of male and female pronuclei, as well as in nuclei of 2- to 8-cell embryos. Some regions appeared densely covered with NPCs, whereas others lacked NPCs entirely (Fig 1A and 1B; S1A Fig–S1E Fig and S2A Fig). After MGA the distribution of NPCs became rather uniform over the entire nuclear envelope (Fig 1C and 1D; S1F Fig—S1I Fig and S2B Fig). NPC clusters, which were indicated by confluence of NUP153 signals, appeared more prominent in the large pre-MGA nuclei compared with the smaller post-MGA nuclei (S2 Fig). Visual inspection of 3D-SIM sections suggested that these nuclei also carried the highest total number of NPCs, but limitations of the resolution of 3D-SIM prevented precise counts of individual NPCs in such clusters.

### NPCs are restricted to parts of the nuclear envelope in contact with DNA

Regions of the nuclear envelope and its associated nuclear lamina, which lacked NPCs in nuclei from embryos up to the onset of MGA at the 8-cell stage, were characterized by a lack of contacts with DAPI-stained DNA (Fig 1A and 1B). In nuclei of post-MGA embryos DNA apparently contacted the entire envelope with its associated lamina in line with a rather uniform distribution of NPCs (Fig 1C and 1D). Fig 1C presents an exceptionally large post-MGA nucleus from a 19-cell embryo. Some features of its phenotype are similar to the nucleus from an 8-cell embryo shown in Fig 1B. These features include the presence of a major lacuna in the nuclear interior, which is apparently free of DAPI-stained DNA and surrounded by major DAPI-stained chromatin bodies, which are separated by wide interchromatin compartment (IC) channels. In contrast to pre-MGA nuclei (Fig 1A), this post-MGA nucleus revealed a thin rim of DAPI-stained DNA associated with the entire lamina and a rather uniform distribution of NUP153. A uniform pattern of attachments of DAPI-stained DNA with the lamina was a characteristic feature of nuclei in post-MGA embryos (Fig 1C and 1D; S1F Fig–S1I Fig). These observations suggest that chromatin contacting the nuclear envelope or its lamina is an essential requirement for the presence of NPCs in the corresponding part of the nuclear envelope.

### Different types of nuclear invaginations during preimplantation development

**Fig 2:**
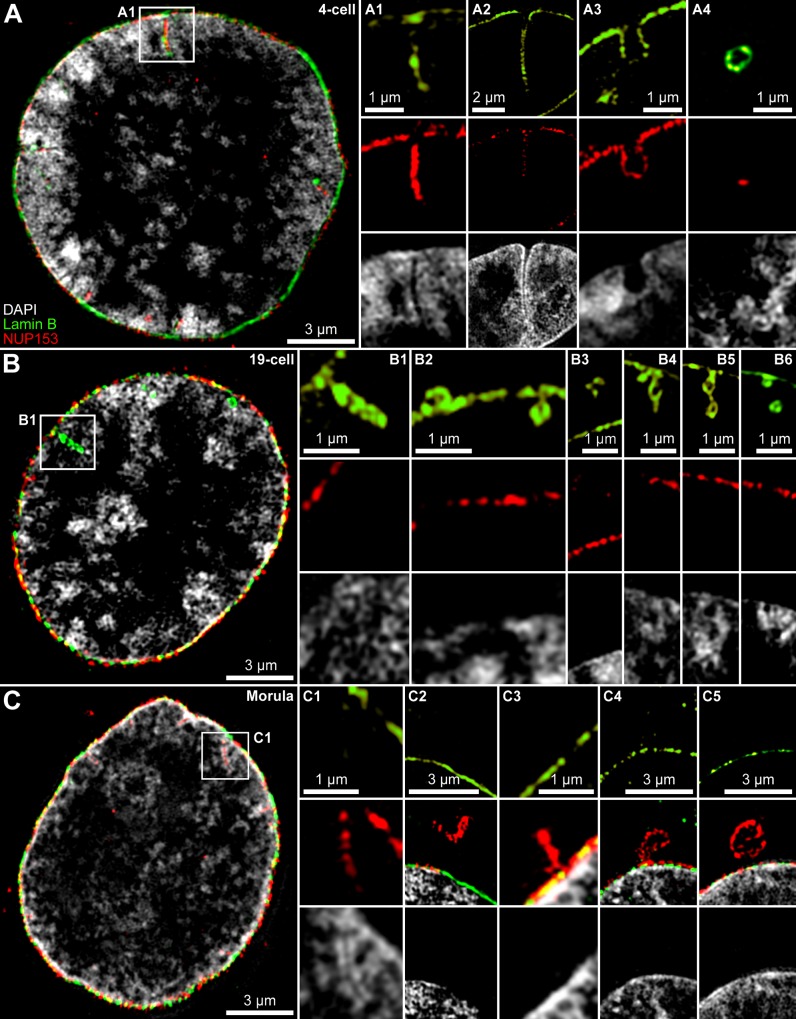
Invaginations, extrusions and excisions of the nuclear envelope/lamina studied by 3D-SIM. **A.** Midsection through a nucleus from a 4-cell embryo with DAPI-stained DNA (grey) and immunostained NUP153 (red) and lamin B (green). **Panel A1.** Enlarged view of the boxed area in A shows a NUP153+/lamin B+ invagination lined by DAPI-stained chromatin. **Panel A2.** Deep NUP153+/lamin B+ invagination recorded from another nucleus. Other sections (not shown) indicate that this invagination formed part of a tunnel connecting opposite sides of this nucleus. **Panel A3.** Wide NUP153+/lamin B+ invagination. **Panel A4.** Vesicle-like, NUP153+/lamin B+ intranuclear structure with sparse NUP153 signal observed in the nuclear interior. **B.** Midsection through a nucleus from a 19-cell embryo. **Panel B1.** Enlarged view of the boxed area in B shows a lamin B positive, but NUP153 negative invagination without a clearly marked border of DAPI-stained chromatin. **Panel B2.** NUP153-/lamin B+ material extruding outward from the nuclear envelope (left) or intruding into the nuclear interior (right). **Panel B3.** Extranuclear NUP153-/lamin B+ segments. **Panel B4-B6.** Intrusions of NUP153-/lamin B+ segments, still connected with the lamina (B4, B5) or entirely disconnected from it (B6; adjacent SIM sections not shown). Notably, invaginations of NUP153-/lamin B+ material were not lined by brightly DAPI-stained chromatin in contrast to NUP153 positive invaginations (compare panels A1, A2). **Panel C.** Midsection through a nucleus from a morula. **Panel C1.** Enlarged view of the boxed area in C shows a NUP153 positive, but lamin B negative invagination surrounded by intensely DAPI-stained chromatin. **Panel C2** presents a NUP153+/lamin B- vesicle in a nucleus from a 2-cell pre-MGA embryo lacking association with the nuclear border (see Fig 4 for a projection displaying vast amounts of similar NUP153-clusters seemingly randomly distributed throughout the cytoplasm). **Panel C3**. Small NUP153+/lamin B- extrusion. **Panels C4** and **C5**. NUP153+ extrusions from the nuclear envelope (C4) forming vesicle-like structures that stay in close proximity with the nuclear border in a 19-cell post-MGA embryo nucleus (C5) (for further details see Fig 4, panels A-E and panels 1–4). S3 Fig presents complete DAPI-, lamin B- and NUP153-stained sections of the nuclei shown in this Figure. Bars: 3 μm for A, B, C, C2, C4, C5; 2 μm for A2; 1 μm for A1, A3, A4, B1-B6, C1, C3.

Multiple types of nuclear invaginations were another striking finding and could be demonstrated throughout the entire preimplantation period (Fig 2; S1 Movie). Three types of invaginations were distinguished by different patterns of immunostaining with anti-NUP153 and anti-lamin B antibodies (Fig 2A–2C). Throughout all stages of preimplantation development 69% of invaginations studied in a total of 90 nuclei were immunopositive for both NUP153 and lamin B (Fig 2A). We refer to them below as NUP+/lamin B+ invaginations. They could be very short and barely recognizable, but could also penetrate deeply into the nucleus or form tunnels.

Eighteen percent of invaginations were immunopositive for lamin B but negative for NUP153 (Fig 2B). These NUP-/lamin B+ invaginations were not lined by intensely DAPI-stained DNA and may not be involved in import/export functions. SIM serial sections yielded frequent examples of NUP-/lamin B+ intranuclear segments and vesicle-like structures inside the nucleus, which were likely derived from NUP-/lamin B+ invaginations (Fig 2B5). Z-stacks did not reveal any contact with the nuclear envelope suggesting their complete isolation (data not shown). NUP-/lamin B+ extranuclear segments or extranuclear vesicle-like structures were rarely noticed. We argue that NUP-/lamin B+ invaginations are part of a mechanism involved in the reduction of the nuclear envelope, which in turn requires a reduction of the lamina beneath the envelope (see Discussion).

Thirteen percent of invaginations were NUP153 positive but lamin B negative (Fig 2C). These NUP+/lamin B- invaginations, as well as NUP+/lamin B+ invaginations, were typically lined by brightly DAPI-stained chromatin (compare Fig 2C with 2A). Large extrusions, as well as vesicle-like structures of NUP153 positive material were noted outside the nucleus up to the 19-cell stage (Fig 2C2–2C5).

### Nuclear volumes decrease during preimplantation development

**Fig 3:**
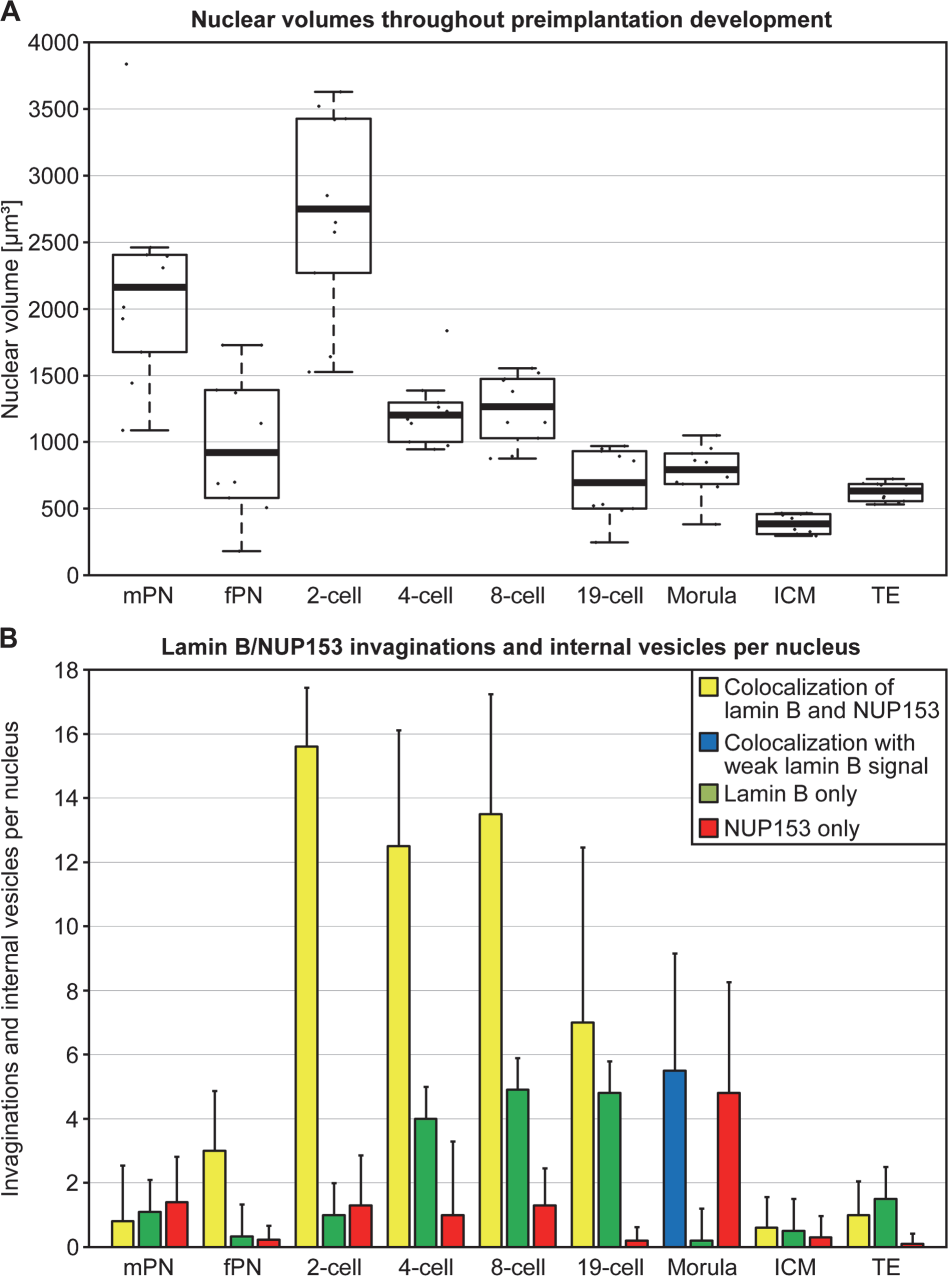
Quantitative evaluation of nuclear volumes (A) and nuclear envelope/lamina invaginations or intranuclear vesicles (B). **A.** Box-/scatterplots represent volume measurements of nuclei at different stages of preimplantation development. 10 volume measurements were performed for each plot. mPN: male pronuclei; fPN: female pronuclei; ICM: blastocyst nuclei from the inner cell mass; TE: blastocyst nuclei from the trophectoderm. **B.** Bars represent average counts of nuclear invaginations and internal vesicles per nucleus with standard errors of the mean from the zygote to the blastocyst stage performed in nuclei after two-color immunostaining of lamin B and NUP153; see box for the meaning of the color-coded columns. For each stage ten nuclei were evaluated. Counts were performed in nuclei from several embryos up to the 8-cell stage and a single embryo each at the 19-cell, morula and blastocyst stages. Note that invaginations and isolated segments were rare events in zygotic pronuclei and blastocyst nuclei compared to the strong, transient increase of invaginations/vesicles in embryos studied from the 2-cell (mGA) to the morula stages.

Nuclear volumes showed a massive decline during preimplantation development (Fig 3A). The largest nuclear volumes were observed at the 2-cell stage, followed by a significant decrease at the 4-cell stage (p <10^–6^). No further significant change was noted between the 4- and 8-cell stages (p = 0.7). A further significant nuclear volume decrease occurred after MGA. Compared with nuclei at the 8-cell stage, nuclear volumes of the 19-cell embryo and morula were significantly smaller (p <0.002; p <0.0005), whereas no significant volume difference was noted between nuclei of these two embryos (p = 1). A further significant decrease was noted between morula stage nuclei and nuclei of the inner cell mass (ICM) (p <0.0001) as well as nuclei of the trophectoderm (TE) of the studied blastocysts (p <0.015). Note that nuclei of the TE were significantly larger than nuclei from the ICM (p <0.0001).

### Quantitative evaluation of nuclear invaginations and internal vesicle-like structures

Fig 3B provides a quantitative assessment of nuclear invaginations described above (Fig 2), and of vesicle-like structures observed in the nuclear interior. Whereas invaginations were rare in pronuclei, they became frequent in embryos from the onset of mGA at the 2-cell stage to the morula stage (about 32 cells). In nuclei of 2- to 19-cell embryos the majority of invaginations were NUP+/lamin B+. After MGA, NUP+/lamin B+ invaginations became less frequent. NUP-/lamin B+ invaginations were most frequently observed between the 4-cell and 19-cell stages. This correlates with a major reduction in nuclear volume and surface (compare Fig 3A). NUP+/lamin B- invaginations peaked at the morula stage. Although a similarly sized fraction of NUP+/lamin B+ invaginations was still observed, the lamin B signal was typically weak. At the blastocyst stage invaginations, intranuclear segments and vesicle-like structures were rarely noted.

### Extranuclear NUP153 positive clusters

**Fig 4:**
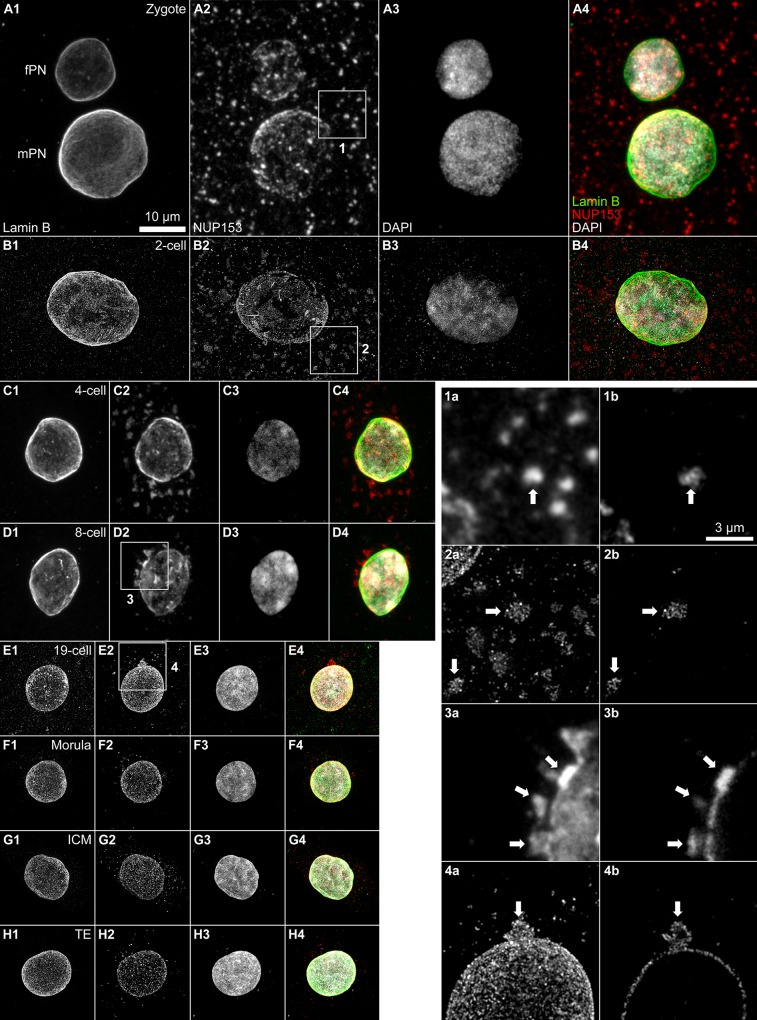
Panels A-H. **Distribution of NUP153 signals during bovine preimplantation development.** The panels show projections of light optical serial sections of nuclei recorded with 3D-CLSM (panels A, C, D) and 3D-SIM (panels B, E-H) at different stages of preimplantation development: pronuclei (panel A), 2-cell stage (panel B), 4-cell stage (panel C), 8-cell stage (panel D), 19-cell stage (panel E), morula stage (panel F), blastocyst stage, inner cell mass (ICM; panel G), blastocyst stage, trophectoderm (TE; panel H). All nuclei are shown with identical magnification (bar: 10 μm). Each panel shows from left to right projection images from (1) lamin B, (2) NUP153, (3) DAPI, and (4) overlay of lamin B (green), NUP153 (red) and DAPI (grey). Deposits of NUP153 clusters were randomly distributed throughout the cytoplasm before the onset of MGA at the 8-cell stage. At MGA and shortly after extranuclear NUP153 clusters were located either at or in close proximity to the nuclear periphery. The clusters were no longer present at the morula stage and later (panels F-H). **1a-4a** show enlargements of boxes around clusters (marked with arrows) presented in A, B, D and E. **1b-4b** show single sections of the same clusters. Bars: 10 μm for A-H; 3 μm for 1–4.

Numerous extranuclear NUP153 positive clusters, located throughout the cytoplasm were noted in nuclei of zygotes, 2-cell and 4-cell embryos (Fig 4A–4C). The distribution pattern of these clusters was markedly changed with the start of MGA in an 8-cell and also in a 19-cell embryo (Fig 4D and 4E). At these stages NUP153 positive extrusions and vesicle-like structures were directly associated with or located very close to the nuclear envelope. No extranuclear NUP153 positive clusters were observed at the morula and blastocyst stages (Fig 4F–4H).

### Distribution of NUP153 in mitotic chromosomes

**Fig 5:**
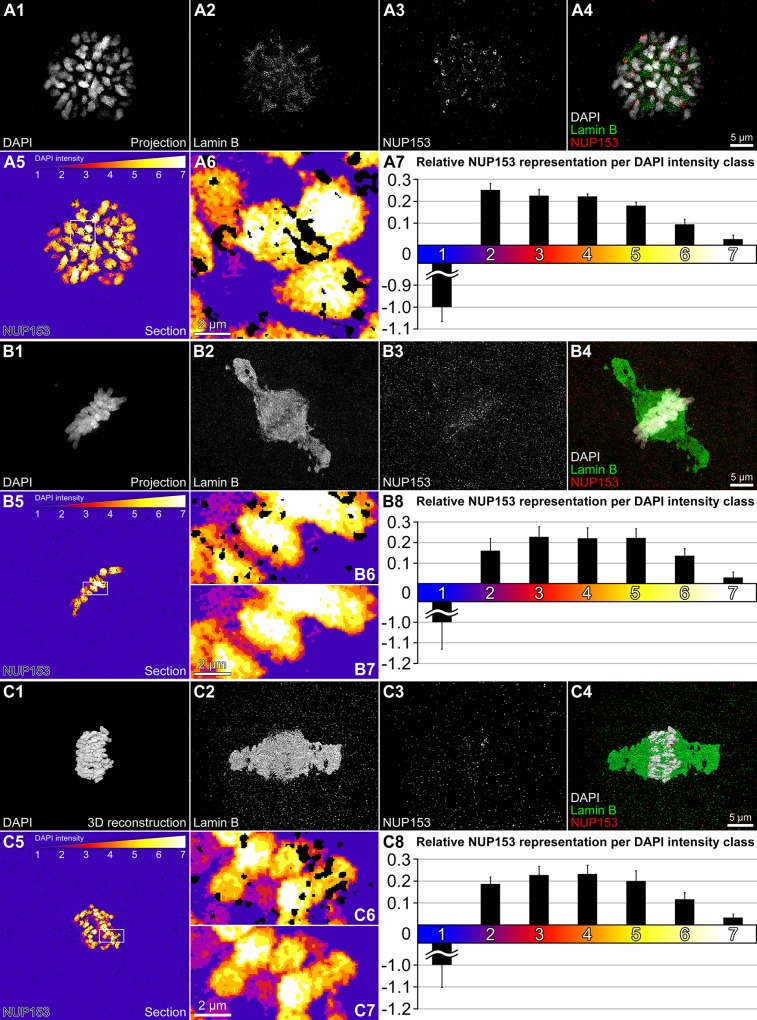
NUP153 topography during mitosis. **A1-A4** and **B1-B4.** Projections of SIM serial sections through metaphase cells of 2-cell embryos stained with DAPI (A1, B1) and immunostained for lamin B (A2, B2) and NUP153 (A3, B3). A4 and B4 show merged images with DAPI (grey), lamin B (green) and NUP153 (red). **C1-C4.** 3D reconstructions of SIM serial sections recorded from an anaphase cell of a 2-cell embryo; DAPI (C1), lamin B (C2), NUP153 (C3), merged image (C4) color-coded as above. 3D reconstructions were rotated in order to provide a view on the two separating anaphase rosettes, which overlapped each other in the original position. Granular lamin B was enriched along the entire mitotic spindle in metaphase and anaphase cells (compare B2 and B4 with C2 and C4). In **A5, B5** and **C5** the metaphase plates (A1, B1) and anaphase rosettes (C1) are presented with seven color-coded DAPI intensity classes. DAPI intensities below the chosen threshold are attributed to class 1 (blue), pixels representing increasing DAPI intensities are color-coded with dark and light red (classes 2 and 3), orange (class 4), dark and light yellow (classes 5 and 6) and white (class 7). NUP153 signals are displayed in black. **A6, B6** and **C6** present enlargements of boxed areas in A5, B5 and C5 with black NUP153 signals. **B7** and **C7** show the same areas as B6 and C6, respectively, without NUP153 signals. A7, B8 and C8 show relative NUP153 accumulations and depletions, respectively, calculated for all seven DAPI classes. In the three mitotic cells the relative numbers of NUP153 positive pixels were significantly enriched in chromatids as compared to the cytoplasm (represented by class 1) (p <10^-15^). NUP153 was mostly overrepresented in the periphery of chromatids (classes 2 and 3). In the innermost area of chromatids (class 7) NUP153 was reduced compared with the chromatin periphery (p <10^-15^), but still significantly enriched in comparison with the cytoplasm (p <10^-15^). Bars: 5 μm for A1-A5, B1-B5, C1-C5; 2 μm for A6, B6 and B7, C6 and C7.

A recent model of postmitotic NPC assembly argues that the NUP107-160 complex assembles at chromatin surfaces and interacts with additional nucleoporins, such as NUP153, for complete NPC assembly [25]. We studied the distribution of NUP153 in DAPI-stained mitoses of bovine 2-cell embryos (Fig 5) together with lamin distributions (Fig 5A1–5A4, 5B1–5B4 and 5C1–5C4). Contrary to large, cytoplasmic NUP153 positive clusters observed during the interphase of 2-cell stage nuclei (Fig 4B2), only small NUP153 particles were distributed throughout the cytoplasm of mitotic cells together with an apparent enrichment in chromatids. We performed a quantitative analysis of this distribution with respect to mitotic chromosomes in ten serial SIM sections of each mitotic cell (Fig 5A5–5A7, 5B5–5B8 and 5C5–5C8). DAPI-stained DNA was segmented into seven classes with equal intensity variance. This approach allowed a threshold-independent signal intensity classification at the voxel level [26]. Class 1 comprises voxels with intensities close to background level, while class 7 represents voxels with highest intensity. We determined both the number of DAPI pixels and the number of positive pixels for NUP153 present in each DAPI intensity class and tested the null hypothesis of a random distribution of NUP153 positive pixels across the 7 DAPI intensity classes. In this case the fraction of NUP153 pixels should be equal to the fraction of DAPI positive pixels representing each class. This null-hypothesis was rejected with a very high probability (p <10^-15^). Fig 5 panels A7, B8 and C8 demonstrate a highly significant, relative accumulation of NUP153 signals over chromosomes (classes 2–7) as compared to a relative depletion in the cytoplasm (represented by class 1). The lowest accumulation of NUP153 was noted in class 7. Our analysis indicates that NUP153 could reach the interior of chromosomes, but localization in areas with the highest chromatin density was apparently less pronounced than at the periphery. Many NUP153 particles were located at the chromosome surface. We expect that these particles carried other components of the disassembled NPCs as well and that their location determined the sites where NPCs were reassembled during the telophase/G1 transition in the reconstituted nuclear envelope.

### Changes of unspliced and spliced *NUP153* RNA during preimplantation development

**Fig 6:**
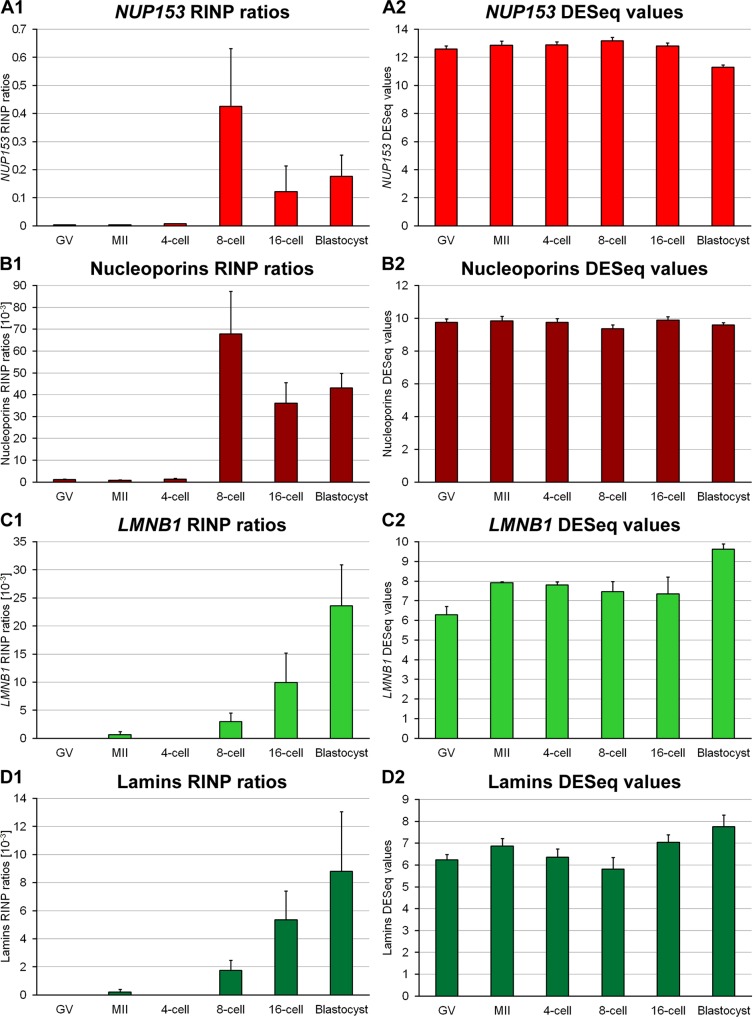
Transcriptome analysis of nucleoporins and lamins. Expression data were generated by Graf et al. [4] and are available at GEO (GSE52415). Data analysis was performed as described [4]. **A1.** Diagram of *NUP153* mRNA ratios of intronic reads to not-covered intronic positions (RINP; see [4]) of three combined replicates for the germinal vesicle (GV) and metaphase II (MII) stages of oocytes as well as the 4-cell, 8-cell, 16-cell and blastocyst stages of embryonic development. **A2.** Diagram of *NUP153* normalized expression values from DESeq for the same stages as shown in A1. The GV and MII stages of oocytes up to the 4-cell stage show a high presence of spliced *NUP153* mRNA compared with a relatively low amount of unspliced, newly synthesized *NUP153* mRNA as shown in A1. Spliced *NUP153* mRNA levels remained stable from the GV to the blastocyst stage (A2). **B1, B2, C1, C2, D1** and **D2** show the diagrams for the same analysis as performed in A1 and A2 for the combined averages of *NUP35*, *NUP37*, *NUP43*, *NUP50*, *NUP54*, *NUP62*, *NUP85*, *NUP88*, *NUP93*, *NUP98*, *NUP107*, *NUP133*, *NUP153*, *NUP155*, *NUP160*, *NUP188*, *NUP205*, *NUP210*, *NUP210L* and *NUP214* (B), *LMNB1* (C) and all lamins (*LMNA*, *LMNB1* and *LMNB2*; D). These results are similar to the results for *NUP153*, indicating that the clustering of NUP153 during interphase is not replicated by lamin B despite its presence in the cytoplasm.

RNA-Seq data were recently generated with the goal to characterize the changing patterns of RNA pools already present in oocytes or synthesized *de novo* after major embryonic genome activation [4]. In the present study these data were further analyzed as described in [4] with respect to maternal and embryonic nucleoporin mRNA and more specifically *NUP153* mRNA. At the germinal vesicle and MII stages of oocytes we noted a relatively large pool of spliced nucleoporin mRNA as demonstrated by a high proportion of exonic and a small proportion of intronic reads (Fig 6A and 6B). Spliced nucleoporin mRNA levels remained relatively stable from the germinal vesicle stage to the blastocyst stage. Initiation of embryonic *NUP43*, *NUP107* and *NUP153* expression was detected at MGA at the 8-cell stage by the presence of paternal SNPs. The same approach detected initiation of embryonic expression of *NUP35* and *NUP160* at the 16-cell stage, and of *NUP88* at the blastocyst stage. While the initiation of embryonic expression could not be statistically confirmed for all nucleoporins, a general trend of a higher ratio of reads in intronic regions vs. not-covered positions in intronic regions (RINP, see [4]) was starting at the 8-cell stage for most nucleoporins (Fig 6B). Embryonic *LMNB1* expression started at MGA as suggested by a significant increase of the RINP value (Fig 6C). While the embryonic expression initiation of *LMNA* coding for lamins A and C and *LMNB2* could not be statistically confirmed, an increase of the RINP value was detected for both genes at the 8-cell stage and later stages as compared to earlier stages (Fig 6D).

## Discussion

The nuclear envelope and its underlying lamina play a central role in normal and disease-associated, pathological higher order chromatin organization [27–29]. Our results shed new light on the mechanisms involved in the distribution of NPCs, the role of nuclear invaginations covered with NPCs in nuclear import and export functions and changes of the nuclear envelope and its underlying lamina to fit massive changes of nuclear size. Current evidence does not suffice to provide a coherent scheme of the remodeling of the nuclear envelope and lamina in nuclei during bovine preimplantation development.

### Contacts of chromatin with the lamina control the insertion of NPCs into the nuclear envelope

Disassembly of the nuclear envelope at the end of prophase and reassembly of its membranes at the end of mitosis from the endoplasmic reticulum (ER) are accompanied by a corresponding disassembly and reassembly of NPCs into sub-complexes and individual nucleoporins [30,31]. In mitotic cells we found most NUP153 distributed as punctuate fluorescent spots throughout the cytoplasm suggesting their presence in small complexes together with other constituents of NPCs or even to some extent as individual proteins. A prominent enrichment of NUP153 was noted at the periphery of mitotic chromosomes. This enrichment suggests the presence of chromatin binding sites for constituents of NPCs, such as nucleoporins. An active localization at specific chromatin sites is indicated by the fact that attachments of chromatin at the lamina-envelope system generally occur in a highly non-random manner via lamina associated chromatin domains (LADs) [32]. Some enrichment of NUP153 was also detected in the interior of chromatids. The reduced number and/or signal intensity of NUP153 in the interior might indicate either a reduced amount of NUP153 or a reduced clustering.

Bas van Steensel and co-workers [33] visualized the fate of LADs in single cells and found that only about 30% of LADs are in intermittent molecular contact with the nuclear lamina. Upon mitosis, LAD positioning was stochastically reshuffled. This observation indicates that the fraction of chromatin connected to the lamina can change from one cell cycle to the next. Another recent study [34] revealed a homogenous folding state of mitotic chromosomes, which was explained by a model based on a linearly organized, longitudinally compressed array of consecutive chromatin loops. In line with these observations we speculate that binding sites for NPC constituents may be distributed throughout a chromatid and that the fraction of these binding sites exposed at the chromatid periphery may change between subsequent mitoses.

Reconstitution of the nuclear envelope starts with tubules or sheet-like structures of double membranes derived from the ER which expand along chromatin surfaces, fuse into larger segments and finally enclose the mass of chromatids to form a complete new envelope [25]. A current model argues that certain NPC constituents at chromatin surfaces mature into NPCs when they become engulfed by growing ER sheets [25]. However, it is not obvious how large areas of the nuclear envelope can be formed without a direct contact with chromatin. At this point we do not know whether the postmitotic daughter nuclei increased in volume only after a complete nuclear envelope had formed around the relatively small volume of closely associated chromatids present at telophase or whether daughter nuclei were very large from the beginning. According to the first scenario the nuclear envelope was able to form along the surface of closely packaged chromatids and expanded only thereafter together with the expanding nuclear volume. Accordingly, lamin B required for the expanding lamina was imported via already existing NPCs. Areas of the nuclear envelope lacking NPCs may indicate that these parts quickly lost contact with chromatin. The second scenario would imply that it is possible to reassemble large parts of the nuclear envelope at sites located initially remote from chromatin.

In addition to the NPCs formed during reassembly of the nuclear envelope, many more NPCs must be assembled during interphase to approximately double their number before the onset of the next mitosis [35]. The mechanisms for the formation of these additional NPCs may differ from the mechanism for postmitotic NPC assembly [36]. Our data indicate that the interphase formation of NPCs in large pre-MGA nuclei occurred only in parts of the nuclear envelope where the corresponding lamina was in direct contact with chromatin, but was absent in parts of the envelope without such contacts. After MGA both contact sites of chromatin with the lamina and NPCs became much more uniformly distributed.

### Role of nuclear envelope remodeling in nuclear volume reduction

Intranuclear envelope segments and vesicles were previously described as intranuclear annulate lamellae in electron microscopy studies of oocytes of numerous species [37,38], as well as in nuclei of mouse preimplantation embryos [39]. Based on our quantitative analysis, we hypothesize that nuclear envelope invaginations without NPCs, as well as isolated intra- and extranuclear segments of the lamina and nuclear envelope, respectively, are part of a mechanism for nuclear envelope reduction during interphase, which accompanies nuclear volume reduction during preimplantation development (interphase hypothesis). As an alternative or in addition to this hypothesis, isolated lamin B and/or NUP153 immunopositive segments and vesicle-like structures may form during mitosis either during the breakdown of the nuclear envelope or during the nuclear envelope reconstitution at late telophase/early G1 phase (mitosis hypothesis). However, both a maximum of NUP153-/lamin B+ invaginations and of NUP153+/lamin B- extrusions from the nuclear envelope were observed in nuclei at the 8-cell stage. Cells at this stage show the longest interphase length of all stages, and consequently the lowest mitotic rate, during bovine preimplantation development [40]. This supports the interphase hypothesis.

### Role of nuclear envelope remodeling in nuclear import/export functions

Our data indicate a particularly high demand for NPCs in embryos preparing for and passing through MGA. During the first cell cycles preceding MGA, such a need may result from the necessity to re-import into newly formed daughter nuclei large amounts of maternally derived proteins or proteins synthesized *de novo* from maternal mRNAs. Invaginations in large nuclei at early preimplantation development covered with NPCs may also help to improve nuclear import and export functions by reducing the average distances of NPCs to chromatin in need of transcription, replication or repair but embedded deep in the nuclear interior [20].

As already noted above (see Results), the clustering of NPCs prevented precise counts of individual nuclear pores even with the improved resolution of 3D-SIM (ca. 100 nm lateral and ca. 300 nm axial). This problem can be overcome by the application of other approaches of super-resolution fluorescence microscopy with still higher resolution (for review see [22]) or alternatively by use of electron microscopy (for review see [41]). One has to take into account that a quantitative determination of NPCs in fixed cells provides only snapshots. New approaches of live cell super-resolution imaging [42] have started to open future avenues for an understanding of the dynamics of NPC formation and distribution. A particularly high need for the synthesis of NPCs was further supported by our RNA-Seq data combined with microscopic observations, which revealed that *de novo* synthesis of nucleoporin mRNAs was initiated at MGA but preceded by large deposits of maternal mRNAs and cytoplasmic NUP153 protein clusters.

### The changing distribution of cytoplasmic NUP153 positive clusters

Our microscopic observations demonstrate the presence of NUP153 positive clusters in the cytoplasm of pre-MGA blastomeres. Such NUP153 aggregates may be part of maternally provided NUP building blocks for the formation of NPCs until *de novo* synthesis of nucleoporins is initiated at MGA. In contrast, NUP153 positive nuclear extrusions and vesicle-like structures exclusively located in close proximity to the nuclear envelope observed at and shortly after MGA may reflect a mechanism of nuclear envelope reduction, which occurs in line with nuclear volume reduction during preimplantation development.

### Import/export functionality of NPC connections with the interchromatin compartment

In the absence of nuclear envelope invaginations, import/export functions are restricted to NPCs except for occasional observations of ribonucleoprotein particle export based on nuclear envelope budding [43]. In an envelope smoothly surrounding the nuclear periphery transcription factors entering the nucleus need to overcome distances of several μm in order to reach specific targets located in the nuclear interior. Considering the functional benefits of nuclear envelope invaginations covered with NPCs [20], one wonders why nuclear invaginations decreased in post-MGA embryos and were rarely detected in blastocyst nuclei and as a more general consideration, why such invaginations did not evolve as a structural feature of nuclei in all cell types and under all functional conditions? For an answer it is important to consider the role of NPCs not in isolation but in context with their connection to the interchromatin compartment (IC) [1,26,44]. The IC starts with channels at nuclear pores [45]. These channels penetrate the layer of dense chromatin beneath the lamina and form a three-dimensionally interconnected channel system in the nuclear interior. The IC occupies the nuclear space between chromatin domain clusters (CDCs), which form the building blocks of chromosome territories [5]. Current evidence demonstrates that essential nuclear functions like transcription, DNA replication and repair occur in the periphery of CDCs, called the perichromatin compartment (PR) [26,41].

The potential functional roles of the interconnected NPC-IC system in nuclear import/export have not yet been fully explored. Several lines of experimental evidence, however, suggest a role of the IC in guiding imported proteins, such as proteins involved in transcription, DNA replication, repair and chromatin modifications, towards chromatin embedded deep in the nuclear interior, as well as for the export of ribonucleoprotein particles carrying messenger RNAs towards NPCs. Mor et al. [46] have described evidence in favor of the hypothesis that interchromatin channels ensure “a steady and continuous wave of mRNPs travelling towards the NPC.” The observation that green fluorescent protein (GFP) diffuses normally within the IC but shows an anomalous diffusion in compact chromatin [47] is in line with the hypothesis that individual proteins may preferentially move within IC channels. This hypothesis is further supported by high resolution fluorescence correlation spectroscopy (FCS) of the mobility of inert monomers, trimers and pentamers of GFP, as well as GFP fusions with other proteins in nuclei of living cells [48]. Larger nuclear particles with a diameter of 100 nm show an obstructed diffusion in distinct corralled regions surrounded by dense chromatin regions [49].

## Conclusions

In conclusion, we argue that the NPC-IC system suffices in nuclei of many cell types to accomplish the needs of nuclear import/export functions even for chromatin embedded deep in the nuclear interior. An additional system of nuclear invaginations covered with NPCs may only be needed in case of extraordinary import/export demands. The case of nuclear envelope remodeling during bovine preimplantation development provides an example for the requirement of nuclear envelope invaginations in the very large nuclei at early preimplantation development but not in the much smaller nuclei at later stages.

## Supporting Information

S1 Fig Changes of nuclear pore distributions during bovine preimplantation development. Midplane sections recorded with confocal laser scanning microscopy (A, B) and 3D-SIM (C-I) from representative nuclei with DAPI-stained DNA (A1–I1), immunostained lamin B (A2-I2) and NUP153 (A3-I3), as well as overlays of DAPI (grey), lamin B (green) and NUP153 (red) (A4-I4). **Panel A**. Male pronucleus. **Panel B**. Female pronucleus. **Panels C-H**. Nuclei from embryos with 2-cells (C), 4-cells (D), 8-cells (E), 19-cells (F), a morula (G) and the inner cell mass (H) and trophectoderm (I) of a blastocyst. Note that lamin B underlines the entire nuclear envelope as a contiguous structure throughout the entire preimplantation development. NUP153, in contrast, is clustered at some regions of the nuclear envelope, but entirely lacking at others in nuclei until the 8-cell stage, i.e. the stage, where MGA occurs. Such regions are denoted by arrowheads in A-E. In post-MGA nuclei a rather uniform distribution of NUP153 was noted throughout the entire nuclear envelope. Bar shown in A1, represents 5 μm for all images.
(TIF)

S2 Fig Nuclear pore and lamin B distributions in embryonic pre- and post-MGA nuclei. **A1-A3.** Top section of a nucleus from a 4-cell embryo immunostained for lamin B (A1) and NUP153 (A2), image overlay (A3; lamin B, green; NUP153, red). Note the strikingly different distribution of lamin B and NPCs with extended lamin B positive areas lacking NPCs in the corresponding part of the envelope. **A4.** Overlay of lamin B signals recorded in SIM serial sections from the nuclear top to the nuclear midplane (half-projection) of the nucleus shown in A1-A3. **A5.** Corresponding half-projection of NUP153 signals from this nucleus. Note large areas free of NUP signals, but filled with lamin B signals in A4. **A6.** Intensity plot of lamin B (black) and NUP153 (red). The area crossed by the plot is marked with a dashed line in A4/A5. The blue lines in A4, A5 and A6 mark the transition from a NUP153 enriched area to a NUP153 depleted area. **B1-B3.** Top section of a nucleus from the 19-cell, post-MGA embryo reveals a more homogeneous distribution pattern: lamin B (B1), NUP153 (B2), image overlay (B3; lamin B, green; NUP153, red). **B4 and B5.** Half-projections of SIM serial sections of lamin B signals (B4) and NUP153 (B5) from a nucleus of the inner cell mass (ICM) recorded in a blastocyst. **B6.** Intensity plots of lamin B (black) and NUP153 (red). The area crossed by each plot is marked with a dashed line in B4/B5.
(TIF)

S3 Fig Midplane sections of the nuclei shown in Fig 2 stained for lamin B (A1-D1), NUP153 (A2-D2) and DAPI (A3-D3) with overlays (A4-D4) of lamin B (green), NUP153 (red) and DAPI (grey). Bar: 3 μm for A-D.
(TIF)

S1 Movie This movie presents a view through SIM serial sections of a typical post-MGA nucleus stained with DAPI featuring multiple nuclear invaginations recorded from a morula stage embryo. (AVI)
