# Supplementary figures and images for: Remodeling of the Nuclear Envelope and Lamina during Bovine Preimplantation Development and Its Functional Implications
Source: PLoS One. 2015 May 1;10(5):e0124619. doi: 10.1371/journal.pone.0124619 (PMC4416817; doi:10.1371/journal.pone.0124619)

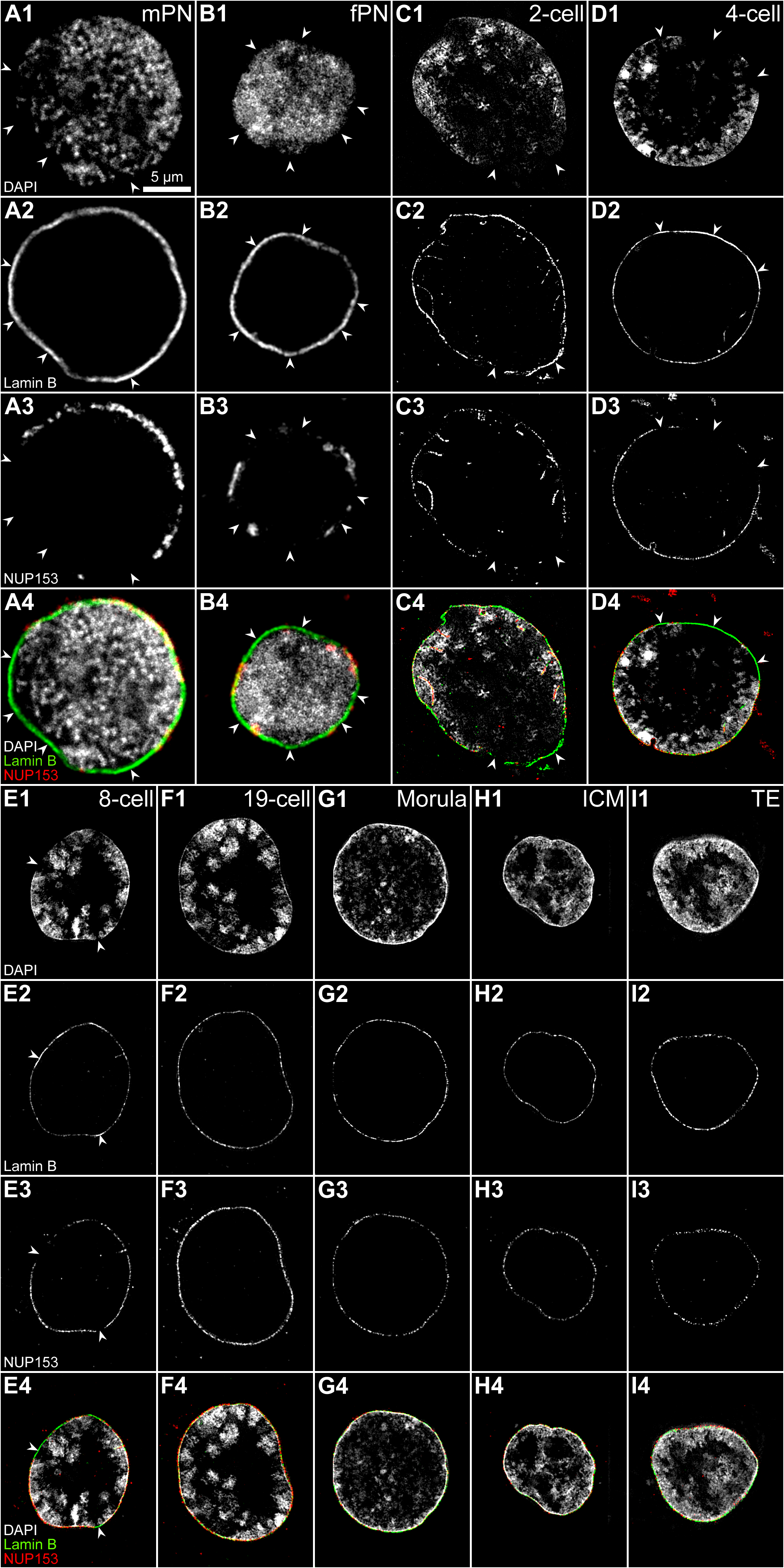

Supplement: S1 Fig — Midplane sections recorded with confocal laser scanning microscopy (A, B) and 3D-SIM (C-I) from representative nuclei with DAPI-stained DNA (A1–I1), immunostained lamin B (A2-I2) and NUP153 (A3-I3), as well as overlays of DAPI (grey), lamin B (green) and NUP153 (red) (A4-I4). Panel A. Male pronucleus. Panel B. Female pronucleus. Panels C-H. Nuclei from embryos with 2-cells (C), 4-cells (D), 8-cells (E), 19-cells (F), a morula (G) and the inner cell mass (H) and trophectoderm (I) of a blastocyst. Note that lamin B underlines the entire nuclear envelope as a contiguous structure throughout the entire preimplantation development. NUP153, in contrast, is clustered at some regions of the nuclear envelope, but entirely lacking at others in nuclei until the 8-cell stage, i.e. the stage, where MGA occurs. Such regions are denoted by arrowheads in A-E. In post-MGA nuclei a rather uniform distribution of NUP153 was noted throughout the entire nuclear envelope. Bar shown in A1, represents 5 μm for all images. (TIF) [file pone.0124619.s001.tif]

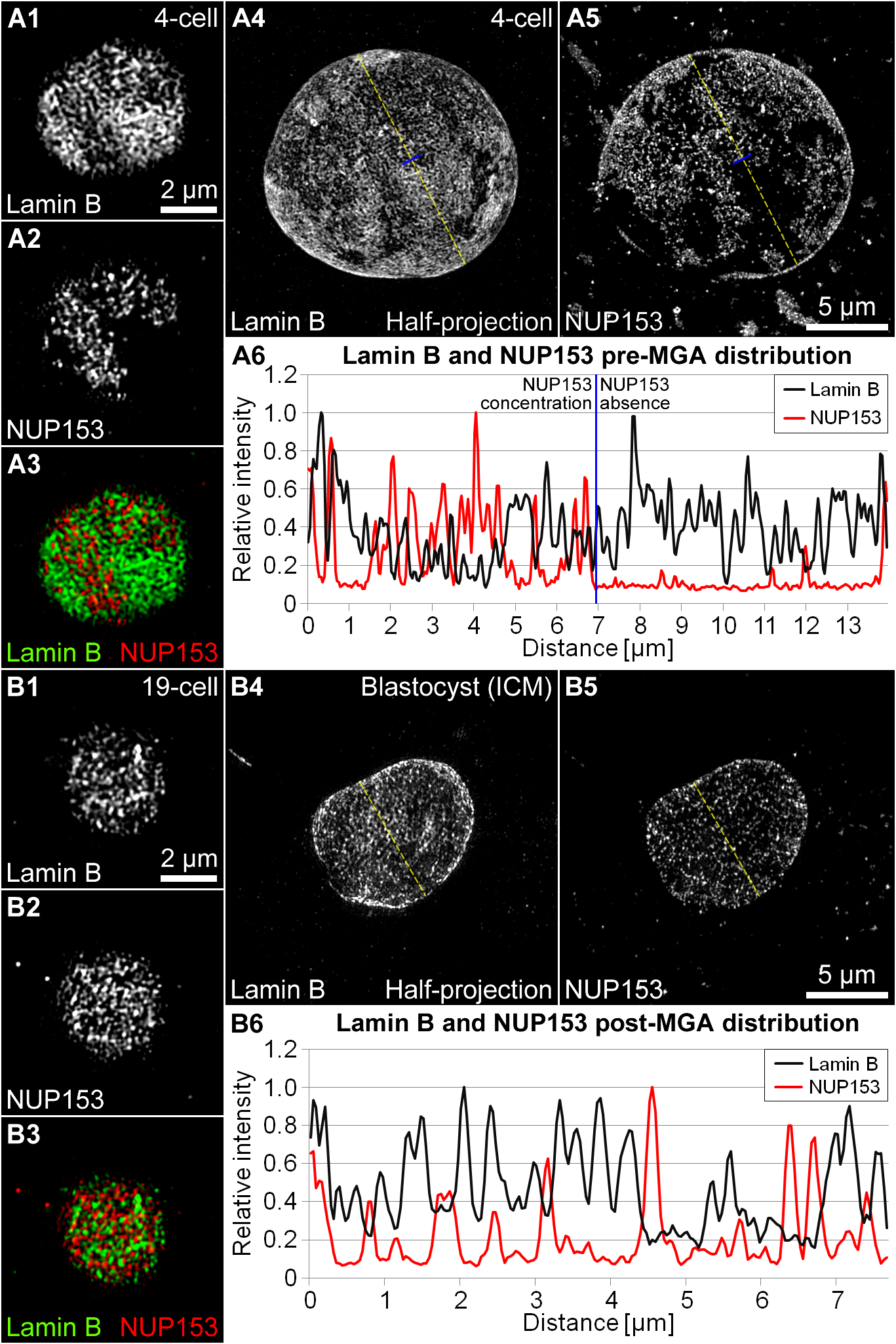

Supplement: S2 Fig — A1-A3. Top section of a nucleus from a 4-cell embryo immunostained for lamin B (A1) and NUP153 (A2), image overlay (A3; lamin B, green; NUP153, red). Note the strikingly different distribution of lamin B and NPCs with extended lamin B positive areas lacking NPCs in the corresponding part of the envelope. A4. Overlay of lamin B signals recorded in SIM serial sections from the nuclear top to the nuclear midplane (half-projection) of the nucleus shown in A1-A3. A5. Corresponding half-projection of NUP153 signals from this nucleus. Note large areas free of NUP signals, but filled with lamin B signals in A4. A6. Intensity plot of lamin B (black) and NUP153 (red). The area crossed by the plot is marked with a dashed line in A4/A5. The blue lines in A4, A5 and A6 mark the transition from a NUP153 enriched area to a NUP153 depleted area. B1-B3. Top section of a nucleus from the 19-cell, post-MGA embryo reveals a more homogeneous distribution pattern: lamin B (B1), NUP153 (B2), image overlay (B3; lamin B, green; NUP153, red). B4 and B5. Half-projections of SIM serial sections of lamin B signals (B4) and NUP153 (B5) from a nucleus of the inner cell mass (ICM) recorded in a blastocyst. B6. Intensity plots of lamin B (black) and NUP153 (red). The area crossed by each plot is marked with a dashed line in B4/B5. (TIF) [file pone.0124619.s002.tif]

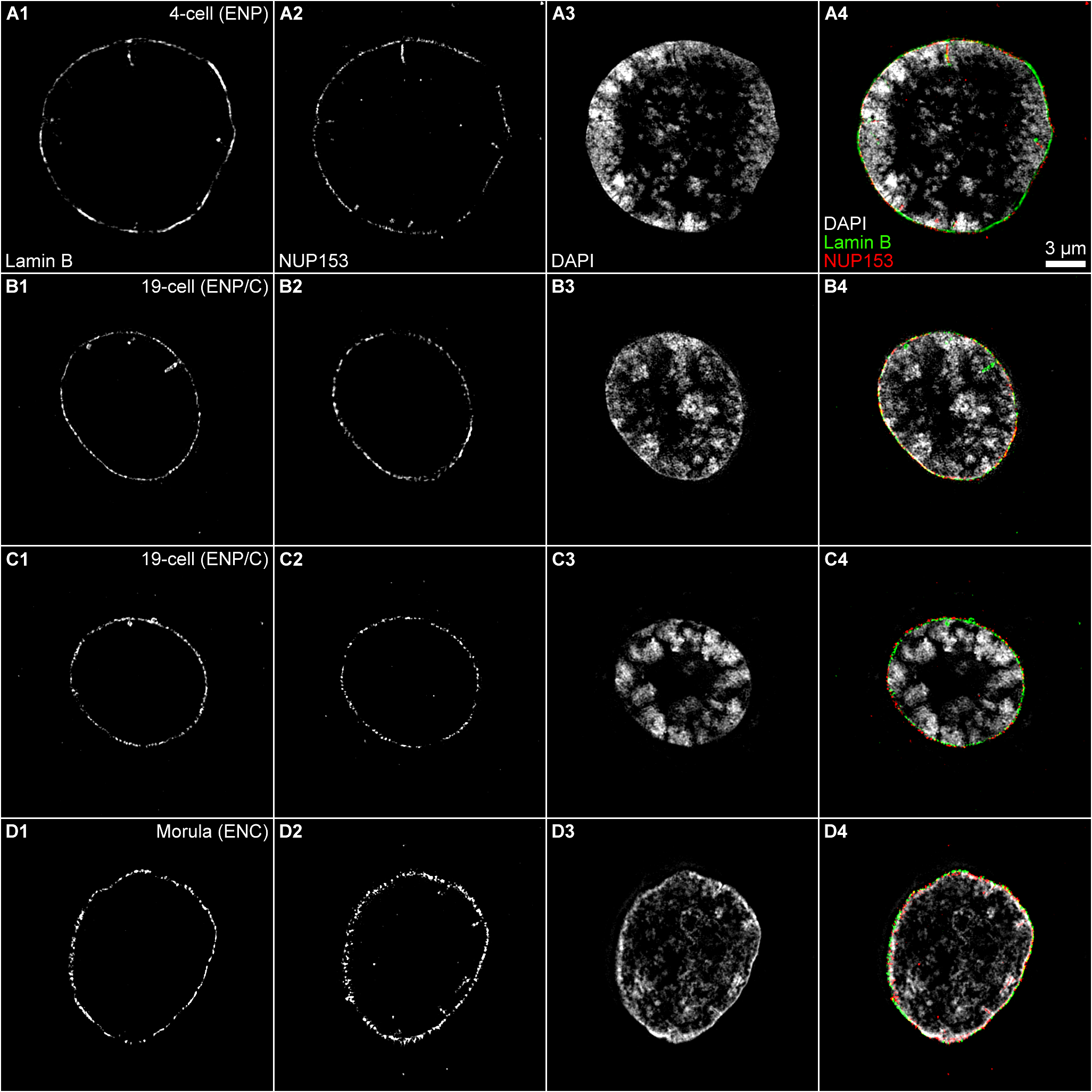

Supplement: S3 Fig — Bar: 3 μm for A-D. (TIF) [file pone.0124619.s003.tif]
